# Older Peer-Delivered and Smartphone-Supported Integrated Medical and Psychiatric Self-Management Intervention

**DOI:** 10.1093/geroni/igaa057.2156

**Published:** 2020-12-16

**Authors:** Karen Fortuna

**Affiliations:** Dartmouth College, Concord, New Hampshire, United States

## Abstract

PeerTECH is older peer-delivered and technology-support integrated medical and psychiatric self-management intervention developed by older adult peer support specialists. Older adult peer support specialists are older adults with a lived experience of a mental health condition, who are trained and accredited to provide support services to others with similar conditions. A pre/post trial by our group has shown PeerTECH is associated with statistically significant improvements in self-efficacy for managing chronic disease and psychiatric self-management skills. This presentation will discuss the feasibility and potential effectiveness of a 3-month pre/post study with older adults with SMI. We will discuss the potential effectiveness of PeerTECH with older adults with SMI related to loneliness, distress, and medical and psychiatric self-management. in conclusion, we will discuss the main and interactive effects of loneliness and factors linked to mortality.

